# Complication Profile of Open Calcaneal Fractures: A Systematic Review of Vascular Injury Reporting and Clinical Outcomes

**DOI:** 10.7759/cureus.109219

**Published:** 2026-05-19

**Authors:** Manahil Awan, Karthika Kalissery Biju Chandrasekhar, Mohammed Elfatih Elbadri, Anwar Al-Kassar, Shashwat Shetty, Nnaemeka C Onyia, Haleema Sadia

**Affiliations:** 1 Trauma and Orthopaedics, University Hospitals Birmingham NHS Foundation Trust, Birmingham, GBR; 2 Trauma and Orthopaedics, The Hillingdon Hospitals NHS Foundation Trust, London, GBR; 3 Orthopaedics, Khor Fakkan Hospital, Emirates Health Services (EHS), Sharjah, ARE; 4 Vascular Surgery, Countess of Chester Hospital, Chester, GBR; 5 Orthopaedics, Hillingdon Hospital, Uxbridge, GBR; 6 General Surgery, NES Healthcare, London, GBR; 7 Surgery, Allama Iqbal Medical College, Multan, PAK

**Keywords:** complications, dorsalis pedis artery, open calcaneal fracture, posterior tibial artery, vascular injury

## Abstract

Open calcaneal fractures are rare, high-energy injuries associated with severe soft tissue damage and a high risk of complications. This systematic review evaluated complication patterns and vascular injury reporting in open calcaneal fractures. A Preferred Reporting Items for Systematic Reviews and Meta-Analyses (PRISMA)-guided search of PubMed, Embase, Scopus, and the Cochrane Library identified four eligible studies, including a total of 455 patients. Data were extracted on vascular injury, infection, amputation, reoperation, and functional outcomes, and a narrative synthesis was performed due to heterogeneity. Posterior tibial artery injury was reported in limited cases, while dorsalis pedis artery injury was not documented. Overall complication rates were high, including superficial infection up to 9.6%, deep infection up to 12.2%, osteomyelitis at 5.2%, and amputation rates between 5% and 8%. One large cohort reported wound-related revision in nearly half of the cases. Vascular assessment was inconsistently reported and rarely supported by imaging. These findings highlight a significant complication burden and a persistent gap in the standardised evaluation of arterial injury in open calcaneal fractures.

## Introduction and background

Calcaneal fractures represent approximately 2% of all fractures and are the most common tarsal bone injury [1]. Open calcaneal fractures are uncommon but are associated with high-energy trauma and severe soft tissue disruption [2]. These injuries frequently result in prolonged morbidity and functional impairment. The calcaneus has a complex vascular network primarily supplied by branches of the posterior tibial and dorsalis pedis arteries [3]. Disruption of these vessels in open fractures can compromise soft tissue viability and increase the risk of infection, non-union, and limb loss. Despite this, vascular injury patterns remain poorly described in the literature.

Management of open calcaneal fractures is challenging and typically involves urgent debridement, intravenous antibiotics, and staged fixation [4]. Reported complication rates remain high, with infection and wound breakdown being the most common adverse outcomes [5]. However, most studies focus on fracture management rather than vascular injury implications. The primary aim of this systematic review was to evaluate complication patterns and vascular injury reporting in open calcaneal fractures. The secondary aim was to identify gaps in vascular assessment and functional outcome reporting within the existing literature.

## Review

Materials and methods

Search Strategy

Table 1 shows that a comprehensive and systematic literature search was conducted in accordance with the Preferred Reporting Items for Systematic Reviews and Meta-Analyses (PRISMA) 2020 guidelines to ensure transparency, reproducibility, and methodological rigour [6]. The search was performed across four electronic databases: PubMed, Embase, Scopus, and the Cochrane Library, from their inception to March 2026. No language restrictions were applied. The search strategy combined controlled vocabulary terms (e.g., MeSH and Emtree) and free-text keywords related to open calcaneal fractures and vascular injury. The core concepts were used: “open calcaneal fracture,” “calcaneus injury,” “hindfoot trauma,” “dorsalis pedis artery,” “posterior tibial artery,” “vascular injury,” “arterial disruption,” and “complications.” Boolean operators “AND” and “OR” were used to combine search terms appropriately. Reference lists of all included studies were also manually screened to identify additional eligible studies. The final search was last updated on April 1, 2026. This strategy was developed to maximise sensitivity for identifying rare vascular injuries associated with calcaneal fractures while maintaining specificity for clinically relevant studies reporting complications and outcomes.

**Table 1:** Search strategy table

Database	Search terms used	Limits applied	Time coverage
PubMed	(“open calcaneal fracture” OR “calcaneus fracture”) AND (“dorsalis pedis artery” OR “posterior tibial artery” OR “vascular injury”) AND (“complications” OR “amputation”)	None	Inception-March 2026
Embase	(‘calcaneus fracture’/exp OR “open calcaneal fracture”) AND (“posterior tibial artery” OR “dorsalis pedis artery”) AND (“vascular injury” OR complications)	None	Inception-March 2026
Scopus	TITLE-ABS-KEY (calcaneal fracture AND vascular injury AND complications)	None	Inception-March 2026
Cochrane Library	calcaneal fracture AND vascular injury	None	Inception-March 2026

*Eligibility Criteri*a

Eligibility criteria were defined using the PICO framework to ensure structured and focused study selection [7]. The Population (P) included adult patients with open calcaneal fractures. The Exposure/Intervention (I) Reported vascular injury or vascular assessment in open calcaneal fractures. The Comparator (C), where applicable, included open calcaneal fractures without documented vascular injury or studies without a direct comparison group, given the rarity of the condition. The Outcomes (O) included post-injury complications such as superficial and deep infection, osteomyelitis, wound complications, amputation rates, reoperation rates, and functional outcomes where available. Studies were considered eligible if they were original human studies (retrospective cohorts or case series) reporting open calcaneal fractures with documented complications and/or vascular injury. Studies were excluded if they were case reports, animal studies, review articles, editorials, conference abstracts, or studies lacking outcome or complication data relevant to the review question.

Study Selection

Study selection was conducted in accordance with PRISMA 2020 guidelines. All identified records were imported into a reference management system, and duplicates were removed prior to screening. Titles and abstracts were independently screened against predefined eligibility criteria. Full-text articles were then assessed for eligibility by applying the inclusion and exclusion criteria. Any disagreements regarding study inclusion were resolved through consensus after re-evaluation of the full text. The final studies included in the review were those meeting all eligibility criteria and containing sufficient data on open calcaneal fractures with reported complications and/or vascular injury. Two reviewers independently screened titles, abstracts, and full texts. Disagreements were resolved through discussion and consensus with senior author review where necessary.

Data Extraction

Data were extracted on reported complications, vascular injury reporting, management strategies, and available functional outcomes, although vascular assessment methods and functional outcomes were inconsistently reported and follow-up duration. Where available, details regarding the severity of injury and surgical intervention were also recorded. All extracted data were verified for accuracy against the original publications.

Risk of Bias Assessment

Risk of bias was assessed using appropriate validated tools based on the study design. Retrospective cohort studies were evaluated using the Newcastle-Ottawa Scale (NOS) [8], while case series were assessed using the Joanna Briggs Institute (JBI) Critical Appraisal Checklist [9]. The overall risk of bias across included studies was predominantly moderate, primarily due to retrospective designs, small sample sizes in some studies, and inconsistent reporting of vascular injury and functional outcomes. Additional limitations included heterogeneity in injury severity, variability in surgical management protocols, and lack of standardised outcome measures across studies.

Data Synthesis

Given the rarity of dorsalis pedis and posterior tibial artery injuries in open calcaneal fractures and the heterogeneity of the included studies, a qualitative narrative synthesis was performed. A meta-analysis was not feasible due to variability in study design, outcome definitions, and inconsistent reporting of vascular injury-specific data. The synthesis focused on summarising complication patterns, vascular injury reporting, management strategies, and functional outcomes across studies. Findings were grouped thematically under vascular injury incidence, infection-related complications, limb salvage outcomes, and reported functional results to allow structured comparison across studies.

Results

Study Selection Process

**Figure 1:**
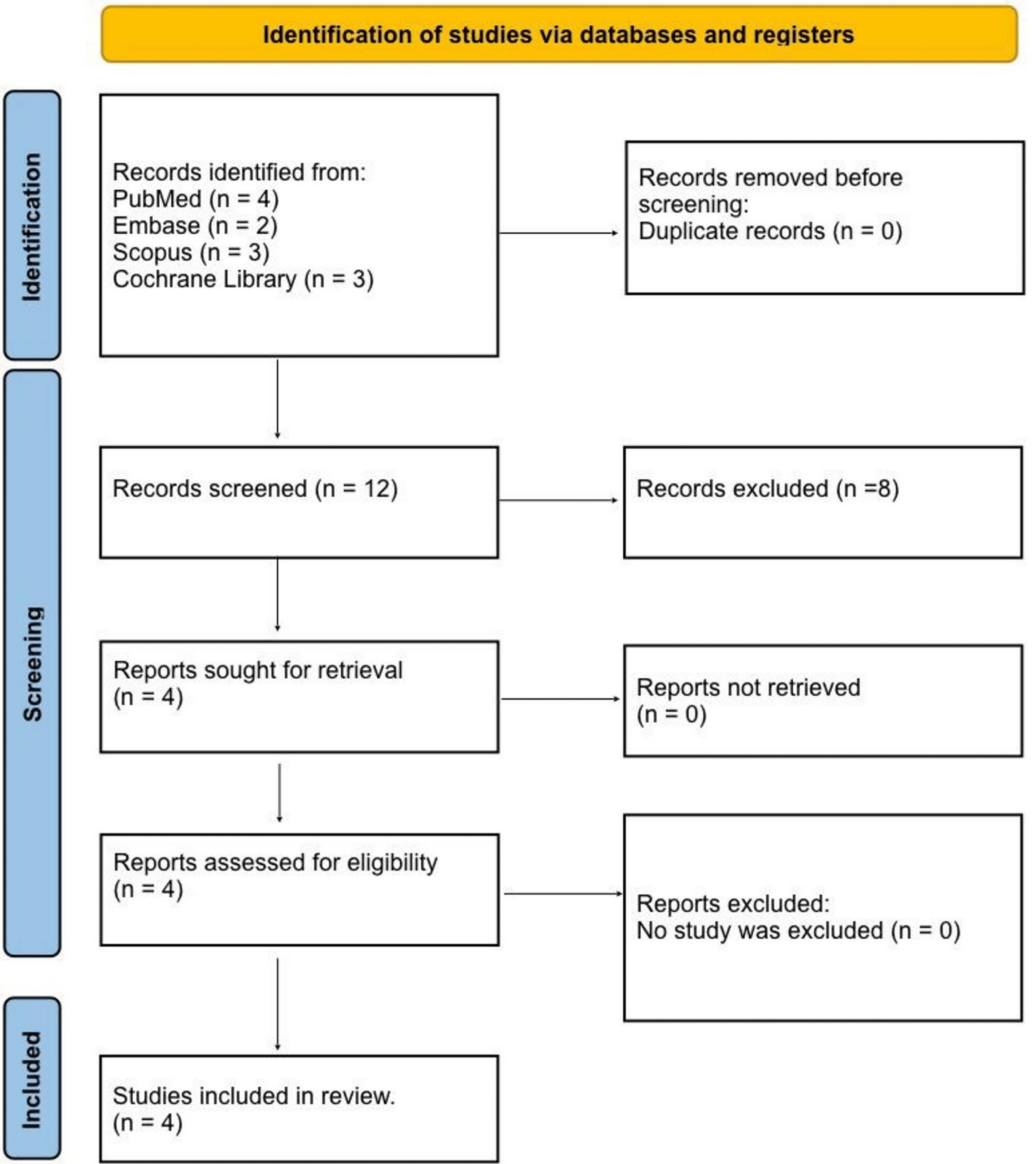
PRISMA 2020 flow diagram PRISMA: Preferred Reporting Items for Systematic Reviews and Meta-Analyses

Figure 1 shows, total of 12 records were initially identified through database searching across PubMed, Embase, Scopus, and the Cochrane Library, as these were found reflecting the requirements of the objectives. After removal of duplicates (n = 0), all 12 records were screened based on title and abstract relevance. Following screening, eight records were excluded as they did not meet the predefined eligibility criteria, leaving four full-text articles for detailed assessment. All four full-text articles were retrieved and assessed for eligibility, with no studies excluded at this stage. Ultimately, four studies met all inclusion criteria and were included in the final qualitative synthesis of this systematic review. No case reports, animal studies, editorials, or conference abstracts were included or excluded at the full-text stage.

Characteristics of the Selected Studies

A total of four studies were included in this systematic review, comprising three retrospective cohort studies and one case series, with sample sizes ranging from 18 to 238 patients. Worsham et al. (2016) evaluated 62 patients with 64 open calcaneal fractures and reported posterior tibial artery transection in four cases, with an overall amputation rate of 8% [10]. Wiersema et al. (2011) included 112 patients with open calcaneal fractures and demonstrated medial wound involvement in 54.8% of cases, with complication rates including superficial infection (9.6%), deep infection (12.2%), osteomyelitis (5.2%), amputation (5.2%), and reoperation (13.9%), with a mean follow-up of 9.1 months [11]. Sturz-Jantsch et al. (2025) analysed 238 patients with 288 calcaneal fractures and reported a wound-related revision rate of approximately 49% following early surgical management, although vascular injury and detailed complication stratification were not specified [12]. Berry et al. (2004) reported on 43 patients with open calcaneal fractures and described high infection and amputation rates in a case series format without uniform quantitative reporting of vascular injury or outcomes [13]. Overall, the evidence demonstrates a consistently high complication burden in open calcaneal fractures, while reporting of dorsalis pedis and posterior tibial artery injuries remains limited and inconsistent across the literature as shown in Table 2. No included study specifically reported dorsalis pedis artery injury.

**Table 2:** Characteristics of included studies n = number of patients, NR = not reported, IV = intravenous, I&D = irrigation and debridement

Authors and year	n	Study design	Level of evidence	Population	Artery injured	Type of vascular injury	Wound location	Management	Superficial infection	Deep infection	Osteomyelitis	Amputation
Worsham et al. (2016) [10]	62	Retrospective cohort	III	Open calcaneal fractures	Posterior tibial artery (4 cases)	Transection	NR	Debridement, antibiotics, fixation	NR	NR	NR	8%
Wiersema et al. (2011) [11]	112	Retrospective cohort	III	Open calcaneal fractures	NR	NR	Medial (54.8%)	IV antibiotics + I&D + delayed fixation	9.6%	12.2%	5.2%	5.2%
Sturz-Jantsch et al. (2025) [12]	238	Retrospective cohort	III	Calcaneal fractures	NR	NR	NR	Early surgery	NR	NR	NR	NR
Berry et al. (2004) [13]	43	Retrospective cohort	III	Open calcaneal fractures	NR	NR	NR	Debridement + fixation	NR	High (qualitative)	NR	Reported

Risk of Bias Assessment

Table 3 shows, total of four studies were included in the risk of bias assessment, comprising three retrospective cohort studies and one case series. The cohort studies by Worsham et al. (2016), Wiersema et al. (2011), and Sturz-Jantsch et al. (2025) were assessed using the Newcastle-Ottawa Scale (NOS) and demonstrated an overall moderate risk of bias, primarily due to their retrospective design, heterogeneity in injury severity, and incomplete reporting of vascular injury and functional outcomes [10-12]. Berry et al. (2004) was evaluated using the Joanna Briggs Institute (JBI) critical appraisal tool and was judged to have a high risk of bias, mainly due to its small sample size, absence of a comparator group, and limited standardisation of outcome reporting [13]. Overall, the evidence base was characterised by methodological limitations inherent to retrospective trauma literature, with variability in reporting of complications and vascular injury contributing to reduced internal validity across studies.

**Table 3:** Risk of bias assessment of included studies NOS = Newcastle-Ottawa Scale, JBI = Joanna Briggs Institute, NR = not reported

Study	Study design	Risk of bias tool	Risk of bias rating	Justification
Worsham et al. (2016) [10]	Retrospective cohort	NOS	Moderate	Retrospective design, limited vascular detail
Wiersema et al. (2011) [11]	Retrospective cohort	NOS	Moderate	Heterogeneous injuries, incomplete vascular reporting
Sturz-Jantsch et al. (2025) [12]	Retrospective cohort	NOS	Low-Moderate	Large sample, but retrospective heterogeneity
Berry et al. (2004) [13]	Retrospective cohort	JBI	High	Small sample, no comparator

Discussion

Open calcaneal fractures are severe injuries typically resulting from high-energy trauma and are frequently associated with significant soft tissue disruption, contamination, and prolonged morbidity. The findings of this systematic review demonstrate that these injuries carry a consistently high complication burden, with infection, osteomyelitis, reoperation, and amputation representing the most frequently reported adverse outcomes across the included studies. Importantly, vascular injury involving the posterior tibial artery was reported in only one study, while dorsalis pedis artery injury was not explicitly documented in any of the included literature, highlighting a significant gap in current orthopaedic trauma evidence. Across the included studies, infection-related complications remained the most common adverse outcome, with superficial infection rates reaching approximately 9.6% and deep infection rates up to 12.2% as reported by Wiersema et al. (2011) [11]. Osteomyelitis, although less frequent, was still clinically relevant at 5.2%, reflecting the vulnerability of open calcaneal fractures to deep-seated infection due to compromised soft tissue envelopes and poor local vascularity. Amputation rates ranged between 5% and 8% across studies, with higher rates observed in more severe injury patterns and polytrauma populations as demonstrated by Worsham et al. (2016) [10]. These findings reinforce that open calcaneal fractures should be regarded as limb-threatening injuries, particularly when associated with vascular compromise.

The role of arterial injury in this context remains underreported but clinically significant. Only Worsham et al. identified posterior tibial artery transection, suggesting that such injuries may be either underdiagnosed or underreported in retrospective trauma literature [10]. Given the dual vascular supply of the foot via the posterior tibial and dorsalis pedis systems, disruption of one or both vessels may significantly impair perfusion and healing potential. However, none of the included studies systematically evaluated vascular status using angiography or standardised vascular assessment protocols, limiting the ability to correlate arterial injury with outcomes such as infection, non-union, or amputation [10,13]. This represents a critical gap in current evidence.

Comparative analysis across studies also highlights considerable heterogeneity in reporting outcomes and management strategies. While early surgical intervention with debridement and staged fixation remains the standard approach, complication rates remain high even with modern protocols. The large retrospective cohort by Sturz-Jantsch et al. (2025) demonstrated a wound-related revision rate of approximately 49%, further emphasising the complexity of these injuries despite advances in surgical care [12]. In contrast, earlier case series such as Berry et al. (2004) reported high infection and amputation rates, reflecting historical treatment limitations but also reinforcing the persistent severity of these injuries across decades [13].

Several limitations of the available literature must be acknowledged. All included studies were retrospective in design, introducing inherent selection and reporting bias [10,11,13]. Only four studies met the inclusion criteria, limiting the strength and generalisability of conclusions. There was significant heterogeneity in injury severity, classification systems, and treatment protocols, limiting comparability between studies. Functional outcomes were sparsely reported and could not be meaningfully synthesised. Furthermore, vascular injury assessment was inconsistently reported, with no standardised use of imaging modalities such as CT angiography or Doppler ultrasonography [10,13]. Functional outcome measures were also largely absent, preventing meaningful assessment of long-term patient-centred outcomes such as return to work or validated scoring systems [12,13]. Future research should focus on prospective, multicentre studies incorporating standardised vascular assessment protocols in open calcaneal fractures. The routine use of angiographic evaluation in high-energy hindfoot trauma may improve the detection of dorsalis pedis and posterior tibial artery injuries. Additionally, future studies should incorporate validated functional outcome measures, limb salvage scoring systems, and long-term follow-up to better define the true burden of these injuries. Establishing a standardised classification system incorporating both osseous and vascular injury patterns may further improve prognostication and guide management strategies.

## Conclusions

Open calcaneal fractures demonstrate a high complication burden, while vascular injury reporting and assessment remain inconsistent in the current literature. Infection, wound-related complications, and amputation remain the most significant adverse outcomes. Vascular injury involving the posterior tibial artery is rarely reported, and dorsalis pedis artery injury is largely absent from current literature. There is a clear lack of standardised vascular assessment in the management of these injuries. Functional outcomes remain poorly defined across available studies. Further prospective research is required to establish uniform diagnostic and treatment protocols to improve outcomes.

## References

[1] Mitchell MJ, McKinley JC, Robinson CM (2009). The epidemiology of calcaneal fractures. Foot (Edinb).

[2] Buckley R, Tough S, McCormack R, Pate G, Leighton R, Petrie D, Galpin R (2002). Operative compared with nonoperative treatment of displaced intra-articular calcaneal fractures: a prospective, randomized, controlled multicenter trial. J Bone Joint Surg Am.

[3] Gupton M, Özdemir M, Terreberry RR (2026). Anatomy, bony pelvis and lower limb: calcaneus. In: StatPearls [Internet].

[4] Jenter M, Lipton GE, Miller F (1998). Operative treatment for hallux valgus in children with cerebral palsy. Foot Ankle Int.

[5] Benirschke SK, Sangeorzan BJ (1993). Extensive intraarticular fractures of the foot. Surgical management of calcaneal fractures. Clin Orthop Relat Res.

[6] Page MJ, McKenzie JE, Bossuyt PM (2021). The PRISMA 2020 statement: an updated guideline for reporting systematic reviews. BMJ.

[7] Schardt C, Adams MB, Owens T, Keitz S, Fontelo P (2007). Utilization of the PICO framework to improve searching PubMed for clinical questions. BMC Med Inform Decis Mak.

[8] Stang A (2010). Critical evaluation of the Newcastle-Ottawa scale for the assessment of the quality of nonrandomized studies in meta-analyses. Eur J Epidemiol.

[9] Munn Z, Barker TH, Moola S (2020). Methodological quality of case series studies: an introduction to the JBI critical appraisal tool. JBI Evid Synth.

[10] Worsham JR, Elliott MR, Harris AM (2016). Open calcaneus fractures and associated injuries. J Foot Ankle Surg.

[11] Wiersema B, Brokaw D, Weber T, Psaradellis T, Panero C, Weber C, Musapatika D (2011). Complications associated with open calcaneus fractures. Foot Ankle Int.

[12] Sturz-Jantsch GD, Winter M, Hajdu S, Haider T (2025). Analysis of calcaneal fracture-related complications-a retrospective chart review. J Clin Med.

[13] Berry GK, Stevens DG, Kreder HJ, McKee M, Schemitsch E, Stephen DJ (2004). Open fractures of the calcaneus: a review of treatment and outcome. J Orthop Trauma.

